# Evaluating privacy-preserving record linkage using cryptographic long-term keys and multibit trees on large medical datasets

**DOI:** 10.1186/s12911-017-0478-5

**Published:** 2017-06-08

**Authors:** Adrian P. Brown, Christian Borgs, Sean M. Randall, Rainer Schnell

**Affiliations:** 1 Centre for Population Health Research, Curtin University, Western Australia, Kent Street, Bentley, Perth, Western Australia, 6102 Australia; 2 University of Duisburg-Essen, German Record Linkage Center, Lotharstr. 65, Duisburg, 47057 Germany

**Keywords:** Medical record linkage, Blocking, Indexing, Private record linkage

## Abstract

**Background:**

Integrating medical data using databases from different sources by record linkage is a powerful technique increasingly used in medical research. Under many jurisdictions, unique personal identifiers needed for linking the records are unavailable. Since sensitive attributes, such as names, have to be used instead, privacy regulations usually demand encrypting these identifiers. The corresponding set of techniques for privacy-preserving record linkage (PPRL) has received widespread attention. One recent method is based on Bloom filters. Due to superior resilience against cryptographic attacks, composite Bloom filters (cryptographic long-term keys, CLKs) are considered best practice for privacy in PPRL. Real-world performance of these techniques using large-scale data is unknown up to now.

**Methods:**

Using a large subset of Australian hospital admission data, we tested the performance of an innovative PPRL technique (CLKs using multibit trees) against a gold-standard derived from clear-text probabilistic record linkage. Linkage time and linkage quality (recall, precision and F-measure) were evaluated.

**Results:**

Clear text probabilistic linkage resulted in marginally higher precision and recall than CLKs. PPRL required more computing time but 5 million records could still be de-duplicated within one day. However, the PPRL approach required fine tuning of parameters.

**Conclusions:**

We argue that increased privacy of PPRL comes with the price of small losses in precision and recall and a large increase in computational burden and setup time. These costs seem to be acceptable in most applied settings, but they have to be considered in the decision to apply PPRL. Further research on the optimal automatic choice of parameters is needed.

## Background

In medical research, information on patients is often scattered across different databases of several data holders. The task of finding records referring to the same person across one or more datasets is, in medical contexts, denoted as *record linkage*. Linking databases is a valuable and cost-effective technique, increasingly used in public health [1, 2], official statistics [3, 4], medical service research [1, 5], pharmacovigilance [6] and demographic research [7]. Applications of record linkage in medical informatics enabled new research on topics such as increased mortality risk after imprisonment [8], increased risk of road traffic accidents after treatments for drug overdoses [9] or mortality for hepatitis C and HIV vs. non-HIV patients [10].

For many research endeavors, linking the information needed would be trivial if a unique personal identifier (PID) is available. However, in many settings, legal and administrative issues prevent the use of PIDs, restricting data linkage to personal identifiers such as names. Since this requires the release of personally identifying information to trusted third parties [11], privacy regulations, such as the HIPAA Privacy Rules [12] or current EU regulations [13], often mandate using encrypted personal information. Standard probabilistic record linkage methods [3] are sometimes unsuitable for methods based on encrypted identifiers.

A number of new record linkage methods have been developed to overcome this problem at a technical level. These methods, known collectively as *privacy-preserving record linkage*, allow linkages using encrypted identifiers. Although no personal identifying information is released by data custodians, record linkage is still possible.

A summary of privacy-preserving record linkage techniques notes that each method differs in its accuracy, maturity, practicality and suitability for large-scale linkages [14]. Few of the available privacy-preserving linkage techniques are suitable for operational linkage units [15].

One notable method for privacy-preserving record linkage utilises *Bloom filters* to enable linkage [16]. The Bloom filters main advantage over many other approaches is that it incorporates uncertainty into matching, allowing the similarity between two fields to be measured (for instance, between two surnames) – a method regularly used in traditional unencrypted record linkage that typically yields high quality. The original Bloom filter approach encodes each field into a separate Bloom filter (a binary vector) which is then compared for similarity using a measure such as the Sørensen-Dice coefficient or Jaccard index. The Dice coefficient of Bloom filter-encrypted identifiers seems to be comparable to the similarity of a Jaro-Winkler comparison on unencrypted identifiers [17]. As encryption occurs on individual fields, standard record linkage procedures can still be used such as blocking (to reduce the comparison space and allow timely linkage to occur) and the assignment of weights to particular fields. Real-world evaluations show similar linkage quality when comparing Bloom filter-based methods with clear-text probabilistic record linkage [15].

Alternate methods of privacy-preserving record linkage using Bloom filters have been developed, with a single Bloom filter composed from many identifiers. Reasons for using only a single Bloom filter for linkage include legal constraints in some jurisdictions [18] and attempts at improving the privacy of the data [19, 20]. A record-level Bloom filter (RBF) combines all fields into a single Bloom filter using the discriminatory power of each field [20]. Fields with a higher discriminatory power are allocated a larger proportion of bits within the RBF, with some bits excluded completely to maximise privacy. Another composite Bloom filter approach uses a basic set of identifiers to produce a cryptographic long-term key or CLK [19]. This was developed as an irreversibly encrypted, anonymous linkage code, that allowed for small typographical errors in the identifiers.

Both of these composite Bloom filter methods have been shown to increase privacy by reducing the chance of a successful, malicious attack [21, 22]. However, the ability of composite Bloom filters to perform highly accurately and efficiently on large real-world data is unknown. As there are no individual fields, indexing (or blocking) methods such as standard Blocking [3] cannot be used without blocking externally on a separate, encrypted identifier. Other approaches to indexing encrypted identifiers, such as the Sorted Neighbourhood Method [23] and Canopy Clustering [24], have been developed, yet neither show optimal performance in all settings [25]. Another recently introduced method using multibit trees has been shown to be very suitable for CLKs, with potential for good quality linkage, and with performance at least as good as other methods on synthetic data [26].

In this paper, we test the accuracy and efficiency of the multibit tree technique on CLKs generated from large real-world medical data, for which the true links (which records belong to the same person) are already known. Testing multibit trees on real-world data is an important step in verifying its viability for linking record-level Bloom filters in public health settings.

## Methods

### Datasets

Ten years of Western Australian (WA) Hospital Admissions data, along with ten years of New South Wales (NSW) Admitted Patient Data were used in this evaluation. For each of these datasets, we had pre-existing and accurate information about which records belonged to which person.

The datasets had been de-duplicated previously (by the WA Data Linkage Branch (WADLB) [27] and the Centre for Health Record Linkage (CHeReL) [28] respectively). De-duplication was undertaken using a variety of methods including exact matching, probabilistic linkage, and intensive clerical review. WADLB and CHeReL employed rigorous manual reviews of created links and a quality assurance program to analyse and review likely errors. These links have been further validated through use in a large number of research projects and published research articles [29], and are used as a ‘truth set’ for linkage quality estimations.

A summary of these datasets can be found in Table 1. The NSW Morbidity data has been separated into public and private hospital data. The private hospital data contains no name information.

**Table 1:** Missing value percentages

Identifier	NSW morbidity (public hospital)	NSW morbidity (private hospital)	WA morbidity
First Name	3%	100%	<1%
Middle Name	54%	100%	41%
Last Name	<1%	100%	<1%
Date of Birth	0%	0%	<1%
Sex	<1%	<1%	<1%
Suburb	<1%	3%	<1%
Address	2%	22%	<1%
Postcode	<1%	3%	<1%
# Records	13810088	6498579	6772949

### Linkage quality metrics

Linkage quality was evaluated using pairwise precision, recall, and F-measure. Precision refers to the proportion of incorrect links found from all the found links and thus provides a measure of false positives. Recall is the proportion of all correct links found, and thus measures false negatives. The F-measure is the harmonic mean between precision and recall, giving a single figure from which we can compare results. These measures are widely used in the record linkage literature [16, 30].

### CLK method

The CLK encryption method is based on the idea of hashing all available personal identifiers into a single structure called a Bloom filter (a binary vector). Each Bloom filters is used as an encrypted linkage key and can then be compared with other keys, resulting in a score which describes how similar the Bloom filters (and thus the personally identifying information) are.

Four different parameter sets were tested, which corresponded to different choices of personal identifiers to combine into each CLK, and are outlined in Table 2. These parameter sets replicate typical blocking and linkage options in traditional record linkage.

**Table 2:** Identifiers used for each parameter set

Identifier	Parameter sets				Average
	Set 1	Set 2	Set 3	Set 4	length
First Name	✓	✓	✓	✓	5
Middle Name	✗	✓	✗	✓	5
Last Name	✓	✓	✓	✓	6
Date of birth	✓	✓	✓	✓	8
Sex	✓	✓	✓	✓	1
Suburb	✗	✓	✓	✗	8
Address	✗	✓	✗	✗	17
Postcode	✗	✓	✓	✗	4

Consistent with the CLK construction method suggested by Schnell et al. [19], each dataset was transformed into four CLK files, one for each parameter set. All CLKs were 1000 bits in length. Each identifier in the parameter set used to make up the CLK (i.e. first name, date of birth, etc.) was converted into unigrams (individual characters) or bigrams (sets of two overlapping characters) with each unigram or bigram hashed 10 times. The modulus of each hash with respect to the Bloom filter was taken, and this position in the Bloom filter set to 1.

Pairs of Bloom filters are compared using the Jaccard, or Tanimoto, similarity. The intersection of the bit positions set to one in both Bloom filters is divided by the union of the bit positions set to one in the two Bloom filters. This results in a similarity score between 0 and 1, where a higher score reflects a greater similarity measure:

$$J(A,B) = \frac{|\mathrm{A} \cap \mathrm{B}|}{|\mathrm{A} \cup \mathrm{B}|} $$

### Security of CLKs and Bloom filters

The desirable property of all Bloom filter-based encryptions is that they are similarity-preserving. This presents security considerations, as this property can be exploited to attack the encryption and potentially reveal personal identifiers. In recent years, several attacks have been published. The first attack, proposed by Kuzu et al. [21], revealed personal identifiers by performing a frequency analysis of individual fields. A discussion on the scope and limitations of the attack is given by Schnell and Borgs [31].

A second attack was devised by Niedermeyer et al. [32] and extended by Kroll and Steinmetzer [33], which focuses on the frequency distributions of the bit patterns of Bloom filters, as well as CLKs. The attack was very successful in decoding CLKs using the double-hashing scheme as proposed in the original publication [16]. However, replacing the double-hashing scheme with full random hashing prevents the attack [31]. Several other hardening techniques have been proposed to make CLKs more resilient against bit-pattern based attacks [31, 34]. For example, using a stable identifier as an additional part of the secret (password) used for encryption is suggested by [32] as a hardening method (salting). Currently, there are no published attacks on such variants of the CLK construction.

### Multibit trees

Searching for similar pairs is computationally expensive. To reduce the search space and thus improve linkage speeds, tree-based structures can be used for blocking. One prominent method is the use of multibit trees, as suggested by Kristensen et al. [35] and suggested for PPRL by Bachteler et al. [36]. Multibit trees show better performance in terms of quality and linkage speed than most current methods, like Canopy Clustering [26], LSH-based blocking [37] or PPJoin [38]. A tree structure is constructed for one record file by finding multiple match bit positions in all Bloom filters where approximately half the records have their bit position set to one, while the other half exhibits a value of zero. Each of these halves are called leaves. This *split-half technique* is repeated until a user-defined minimum number of records in each leaf is reached (usually one to eight records). For our experiments, a leaf limit of one was used.

To find similar pairs in terms of Tanimoto-similarity, every record in the second dataset is queried sequentially. For each record, an upper bound of the Tanimoto-similarity can be estimated before the actual similarity calculation, by comparing the values at the bit positions of each leaf in the tree. Leaves with a similarity under a user-defined Tanimoto threshold are disregarded in the calculation of the similarities. This way, the search space can be reduced drastically.

For our de-duplication linkages, the same dataset was used for the multibit tree and for the sequential queries. We applied a construction method for multibit trees similar to Bachteler et al. [36], testing multiple Tanimoto thresholds for each parameter set.

### Evaluation strategy

All NSW and WA datasets were encrypted into CLKs for each parameter set as described above. For testing of linkage quality and blocking ability on data with few missing values, the WA CLK dataset was then de-duplicated, using multibit trees as the blocking method, at a range of Tanimoto thresholds. For testing of linkage quality on data with many missing values, a random sample of 5 million records was taken from the combined NSW CLK datasets using parameter set 1 (first name, last name, date of birth and sex). This represents a reasonable sample size for a real-world operation, the name identifiers resulting in approximately 30% missing values. The pair-wise precision, recall and F-measure scores were calculated by comparing results to the ‘truth set.’

For testing of performance, the NSW (Public Hospital) and WA CLK datasets were combined for a dataset with a total of approximately 20 million records. From this combined dataset, random samples were taken to create datasets of 5, 10 and 15 million records. All of these datasets were then de-duplicated, using multibit trees with a single Tanimoto threshold of 0.85, as this has previously been shown to be a reasonable value for most applications [26]. The execution time of the multibit tree search was recorded.

All de-duplication linkages used multibit trees with a leaf limit value of one. The multibit tree outputs all candidate pairs, where the criterion for a pair is that it exceeds the given Tanimoto threshold value.

The evaluation was run on a Windows Server 2012 R2 Virtual Machine, running under ESXi on a Cisco UCSC-C240-M3S Server with Intel Xeon CPU E5-2609@2.40GHz. The VM was assigned 48GB RAM and 6 vCPUs. The evaluation code was assigned 4 vCPUs.

## Results

### Linkage quality

**Fig. 1:**
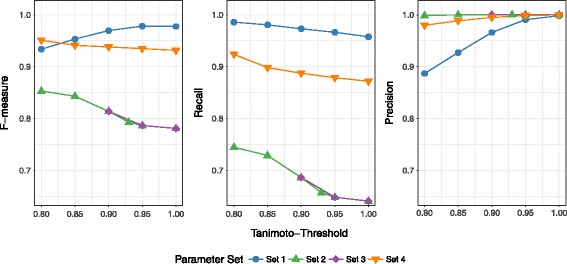
Linkage quality results for WA CLK de-duplication

Results for the de-duplication of the WA CLK dataset can be found in Fig. 1. The highest recall value across all threshold levels was achieved using parameter set 1 (first name, last name, date of birth, sex), with the best value of 0.986 at a threshold of 0.8. The next highest recall was achieved using parameter set 4 (first name, middle name, last name, date of birth, sex). The two lowest recall values came from the use of parameter sets 2 and 3. Values for parameter set 3 at Tanimoto thresholds 0.8 and 0.85 are not provided as these runs failed to complete successfully.

Maximum F-measure varied considerably across the different parameter sets. Highest F-measure was 0.978 from parameter set 1 while lowest F-measure was 0.781 for parameter set 3. The inclusion of address information (parameter sets 2 and 3) tended to reduce overall scores. This can be explained by the varying recall: including addresses introduces unstable identifiers, which either differ in the datasets (e.g. because individuals have moved to a different address) or are missing. This will lead to a reduction in the amount of true pairs found, which is why sets 1 and 4 show superior linkage quality with respect to recall.

All parameter sets but set 1 show high precision scores. Since adding middle names allows for better discrimination between records that would otherwise exhibit the same values across all identifiers, the amount of false positive classifications will decrease, leading to increased precision values for these parameter sets.

The de-duplication linkage of the 5 million sample CLK dataset of the combined NSW Public and Private Hospital datasets (30% of all rows had missing name identifiers) was abandoned after 2 weeks of elapsed execution time. Analysis of the pairs created to that point showed that the number of missing identifiers in the CLKs was leading to the creation of an inordinately large number of false positives; a large portion of rows with only values for date of birth and sex appeared to be linking to each other. The anticipated poor linkage results and excessive processing time led to the decision to abandon all linkage quality tests with this particular dataset.

### Performance

**Fig. 2:**
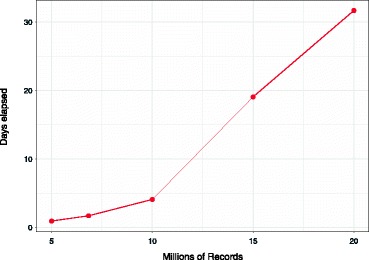
Performance results for combined CLK dataset de-duplication

The time taken to complete the de-duplication of the samples of our combined dataset was a monotone function of the sample size (see Fig. 2). The smallest sample of 5 million records took just under a day to complete. For the large dataset sizes, the run time slowed considerably, taking one month to complete the 20 million de-duplication linkage.

The results in Fig. 2 include the time taken to run the de-duplication of the WA CLK dataset (6.8 million records) was 2,445 minutes. When the same dataset was split into ten roughly equal parts with blocking on year of birth, the total time taken to de-duplicate was 1,828 minutes.

## Discussion

Overall, the use of CLK with multibit trees for a full linkage was not as high quality as could be achieved using either unencrypted linkage or with field level Bloom filters [16]. Using the same dataset (WA Hospital), both unencrypted and field level Bloom filters had achieved an F-measure of 0.99 [15], while this measure achieved a maximum F-measure of 0.978 in our current evaluation. Overall, this difference is small, and this may be acceptable, particularly in cases where the use of a single data item for anonymous linkage is prescribed by law [18].

Our results show that the use of multibit trees for indexing/blocking of CLK data has great potential. The best recall was achieved using parameter set 1, with a value of 0.9858 at a threshold of 0.8. The unencrypted linkage on the same dataset, mentioned previously, had a recall of just 0.981, using standard blocking. The worst results for recall were for parameter sets 2 and 3, with values at all thresholds below 0.75. This is unacceptably low for any linkage, but the inclusion of all identifiers, especially with volatile address information, precludes the ability to match individuals that have changed their address. This shows, that while including more identifiers in the CLKs will usually increase the discriminative power, leading to higher precision, stable identifiers without missing data fields are needed in order to avoid sacrificing recall. While using multibit trees for indexing of CLK data has the ability for a very high coverage of possible links, its quality is ultimately determined by the identifiers used to create the CLK and the quality of the data.

In terms of performance, the linkages were reasonably slow. While operational linkages are commonly performed on an ad-hoc basis, and there are tight processing deadlines to meet, linkages which take more than a few days processing time are probably not feasible. As such, the multibit tree method, as it is currently implemented, could not be recommended for large-scale linkages. As a comparison, an unencrypted linkage of the same 20 million records can be completed within a day.

An alternate approach to using the multibit tree method may be to create a set of hashed blocking variables alongside the CLK, referred to as external blocking [26]. Our simple external blocking of the WA CLK dataset into just ten blocks based on year of birth was enough to reduce the execution time by 25%. In practice, the external blocking required to maintain linkage quality is likely to be more complex, requiring additional information alongside the CLK and may provide an additional attack vector for a malicious individual. However, external blocking provides a considerably faster method for linkage with CLKs, and at this time is a practical way for large-scale private record linkage.

## Conclusion

Further testing is required to improve the CLK linkage results. One factor which is likely to improve results is the use of methods of weighting different personal identifiers based on how likely they are to identify an individual. The impact that a field has within a Bloom filter is directly proportional to how many bits that field encodes. However, in this paper, we used the baseline approach, where the number of bits was solely based on the number of bigrams in the identifier. For example, addresses usually contain many bigrams but are far less useful in identifying an individual over time when compared to date of birth or name. Testing Bloom filters which weight individual fields (by hashing bigrams more or less often) according to their usefulness in identifying individuals (discriminating power) may be an important avenue of further research.

The results reported here are heavily dependent on parameter settings. For these methods to be useful in practice, where ‘truth sets’ are usually not available, tried and tested parameter settings that are robust across different kinds of datasets are required. Missing values were also shown to be a major factor affecting the quality of the indexing and linkage. Since CLKs do not account for the number of identifiers for which valid information is present, calculation of similarities based on CLKs will be attenuated by asymetrically missing identifiers. However, handling missing identifiers in PPRL is a largely unexplored field of research.

Demand for privacy-preserving record linkage is increasing [39]. Security of PPRL solutions against cryptographic attacks is therefore of utmost importance in medical settings.

However, very few techniques for PPRL suitable for large data sets are available. One of these few techniques are Bloom filter-based methods for PPRL. These methods are increasingly used for a wide variety of medical research projects, such as linking mammography data [40] or building a national perinatal database [41]. State of the art variants of Bloom filter-based methods have been shown to be more resilient than competing approaches [31]. Successful attacks on these variants seem to be harder than the effort which can be expected willingly to be provided by a rational attacker [42]. Further hardening Bloom filters is subject of ongoing research by our group.

## Abbreviations

CLK: Cryptographic long-term key
CHeReL: Centre for Health Record Linkage
EU: European Union
HIPAA: Health Insurance Portability and Accountability Act
NSW: New South Wales
PPRL: Privacy-preserving record linkage
PID: personal identifier
RBF: Record-level Bloom filter
WA: Western Australia
WADLB: WA Data Linkage Branch
